# Antioxidant Intake and Biomarkers of Asthma in Relation to Smoking Status—A Review

**DOI:** 10.3390/cimb45060324

**Published:** 2023-06-10

**Authors:** Naser A. Alsharairi

**Affiliations:** Heart, Mind & Body Research Group, Griffith University, Gold Coast P.O. Box 4222, QLD, Australia; naser.alsharairi@gmail.com

**Keywords:** asthma, antioxidant, vitamins, minerals, supplements, biomarkers, oxidative stress, inflammation, smokers, nonsmokers

## Abstract

Asthma is considered a chronic inflammatory disorder associated with airway hyperresponsiveness (AHR). Increased oxidative stress (OS) is a clinical feature of asthma, which promotes the inflammatory responses in bronchial/airway epithelial cells. Smokers and nonsmokers with asthma have been shown to have increases in several OS and inflammatory biomarkers. However, studies suggest significant differences in OS and inflammation biomarkers between smokers and nonsmokers. A few studies suggest associations between antioxidant intake from diet/supplements and asthma in patients with different smoking status. Evidence is lacking on the protective role of antioxidant vitamin and/or mineral consumption against asthma by smoking status with respect to inflammation and OS biomarkers. Therefore, the aim of this review is to highlight current knowledge regarding the relations between antioxidant intake, asthma, and its associated biomarkers, according to smoking status. This paper can be used to guide future research directions towards the health consequences of antioxidant intake in smoking and nonsmoking asthmatics.

## 1. Introduction

Smoking is regarded as a significant risk factor for asthma progression [1]. The number of asthma deaths due to smoking in 2019 was higher in men than in women [1,2]. Asthma is characterized by airway hyperresponsiveness (AHR) and reversible airflow obstruction, which is attributed to increased airway smooth muscle (ASM) contraction [3,4,5]. Asthma is associated predominantly with mast/CD4^+^ cells, T lymphocytes and eosinophils. Mucous hypersecretion, luminal obstruction, goblet cell hyperplasia, and thickening of bronchial walls are commonly observed features in asthma [3].

Tobacco smoke is associated with reduced lung function measured as forced expiratory volume in 1 s (FEV_1_) and increased bronchial hyperresponsiveness in smokers with asthma [6]. Asthmatic patients who smoked ≥10 pack/year had a rapid decline in FEV_1_ and forced vital capacity (FVC) compared with those who smoked <10 pack/year after 12-year follow-up [7]. Secondhand smoke (SHS) exposure has been linked to asthma risk in active and/or former smokers [8]. Exposure to SHS in public places was associated with a marked decrease in peak expiratory flow rate (PEFR) and FVC in asthmatic smokers [9]. The risk of asthma among nonsmokers who were exposed to SHS has increased in a large adult-onset asthma population with 16 years of follow-up [10].

Tobacco smoke consists of a range of toxic chemicals (e.g., benzopyrene, acrolein, crotonaldehyde, phenols, ammonia, nitrosamines, hydrocarbons, aromatic amines), which are potentially harmful to human bronchial epithelial cells (HBECs), causing airway inflammation by increasing mitochondrial reactive oxygen species (ROS) and pro-inflammatory interleukin (IL)-8 cytokine production [11,12]. Downregulation of microRNAs in lung fibroblasts of smokers may affect its function due to aberrant DNA methylation at specific sites [13]. Moderate asthma was associated with lung inflammation, and this response is related to reduced expression of microRNA target genes such as I-miR-146a [14]. Cigarette smoke extract (CSE) exposure in HBECs results in increased oxidative stress (OS) and pro-inflammatory cytokines IL-6, IL-8, and tumor necrosis factor α (TNF-α) by the activation of several inflammatory signaling pathways, including the transcription factor-kappaB (NF-κB), extracellular signal-regulated kinases (ERK 1/2), c-Jun N-terminal kinase (JNK), and mitogen-activated protein kinases (MAPKs) [15]. Tobacco smoke alters immune responses in the lung, triggering asthma by activating Toll-like receptors (e.g., TLR-2 and TLR-4)-stimulated pro-inflammatory cytokine production and increasing total serum immunoglobulin E (IgE) levels in airway epithelial cells [16]. In asthmatic patients, exposure to environmental tobacco smoke (ETS) results in oxidant/antioxidant imbalance, which leads to increased pro-inflammatory biomarkers as assessed by increased TNFα, IL-6, and IL-8 [16]. Evidence suggests that nicotine is not carcinogenic, but it may affect the airway epithelial cells of asthmatic smokers by activating nitrosamine 4(methylnitrosamino)-1-(3–pyridyl)-1-butanone (NNK), which binds to the α7 nicotinic acetylcholine receptor (α7nAChR), leading to AHR and inflammation by upregulating the α7nAChR-mediated signaling pathways [17].

The genetic variants–tobacco smoke exposure interaction has been shown to increase asthma risk in smokers and nonsmokers. Evidence of the interaction between variants of rs9969775 on chromosome 9, rs5011804 on chromosome 12, and active tobacco smoking was reported in asthmatic adults [18]. Genetic variants of NLR Family CARD Domain Containing 4 (NLRP4) inflammasome are implicated in asthma exacerbation in current and former adult smokers as evidenced by high genotype-specific expression of rs16986718G [19]. The presence of mutant AG/GG genotype for *CD14* rs2569190 and rs13150331 (TLR) polymorphism in asthmatic adult smokers increases the risk of the disease [20]. Asthmatic nonsmokers carrying allele homozygotes of rs1384006 C  >  T of the OS responsive kinase 1 (*OXSR1*) gene are at higher asthma exacerbation risk than asthmatic smokers [21].

Few studies have evaluated evidence-based treatment for asthma in smokers. Pycnogenol^®^, a herbal dietary supplement-based extract manufactured by Horphag Research (Geneva, Switzerland) and derived from French *Pinus pinaster* bark, is regarded as an option for the treatment of asthma when used in combination with the inhalation corticosteroid (ICS) therapy, resulting in improvement of asthma symptoms [22]. Asthmatic smokers have ICS insensitivity as compared to asthmatic nonsmokers and are less responsive to the benefits of ICS treatment alone. Alterations of inflammatory phenotypes and glucocorticoid receptors and the reduction of histone deacetylase (HDAC) activity are considered potential mechanisms of corticosteroid insensitivity in asthmatic smokers [23,24]. The combination of ICS therapy and a long-acting β2 adrenergic (LABA) displays a better clinical improvement for smoking and nonsmoking asthmatics than using ICS alone [25,26]. The use of nicotine replacement therapy, varenicline or bupropion, may significantly improve lung function and AHR in asthmatic smokers [25].

There is still a significant amount of uncertainty in the safety and efficacy of dietary supplements for the treatment of lung diseases among smokers and/or nonsmokers due to the limited number randomized controlled trials (RCTs) [27,28]. Thus, there is a need to focus on the role of antioxidants in smoking-related asthma risk. A recent review investigating the effects of dietary antioxidant intake on lung cancer (LC) risk among smokers and nonsmokers suggests that dietary vitamins (C, D, E, and carotenoids) and minerals (zinc and copper) may exert protective effects against cigarette smoke (CS)-induced OS and/or inflammation. However, dietary retinol and iron intake did not provide any protection, and research suggests caution in recommending these for LC treatment [29]. There is a direct association between LC and asthma in smokers [30,31]. Given that smoking is considered a risk factor for asthma through increased levels of OS and inflammatory cytokine production [17], targeting dietary/supplement-derived antioxidants might help our understanding of their role in protecting bronchial epithelial cells against CS-induced-OS/inflammatory biomarkers in smokers and nonsmokers. This paper explores this connection to gain insight into the health consequences of antioxidant consumption and makes recommendations for future studies. To date, there have been no reviews to evaluate antioxidant intake and the biomarkers of OS and inflammation in asthma, according to smoking status.

## 2. Methods

A literature review in the PubMed/MEDLINE database and Google Scholar was conducted for English language studies published between 1 January 2000 and 30 April 2023. The following search terms were used: asthma, diet, supplements, antioxidant, vitamins, minerals, OS, lipid peroxidation (LP), inflammation, biomarkers, antioxidant/oxidant enzymes, bronchial/airway epithelial cells, smoking, smokers, and nonsmokers. Studies focusing on the chronic obstructive pulmonary disease (COPD) were excluded, as the diagnostic biomarkers in asthma are different from both COPD and the asthma-COPD overlap. All studies relevant to the search terms were included, and the search was not restricted to a particular study design.

## 3. Antioxidant Intake and Asthma in Relation to Smoking Status

Studies investigating the associations between antioxidant intake and asthma according to smoking status are limited. Smokers with low dietary vitamin C (VC) intake had chronic bronchitis symptoms associated with asthma compared with those who had higher intake [32]. According to quartiles of carotenoid dietary/supplement intake (carotene, lycopene, and lutein with zeaxanthin), the risk of asthma was reported to be lower in the fourth quartile (≥165.59 μg/kg per day) than the first quartile (<41.43 (μg/kg per day) among current smokers, ex-smokers, and nonsmokers with asthma [33]. One trial revealed no effects of 6 weeks of supplemental vitamin E (VE) on AHR in nonsmokers with asthma [34]. Supplementation with selenium (Se) had no significant improvement in asthma-related quality of life (QoL) and lung function regardless of smoking status [35]. These findings suggest that dietary VC and carotenoids intake may reduce asthma in smokers and/or nonsmokers. Supplementation with VE and Se had no effect on asthma in smokers and nonsmokers. The associations between antioxidant intake and asthma risk according to smoking status are summarized in Table 1.

## 4. Biomarkers of OS and Inflammation in Relation to Smoking Status

OS is regarded as the major contributor to CS-induced airway inflammation [36]. Evidence from many studies, mostly derived from case-control design, has shown that CS activates OS by augmenting airway inflammation in smoking and nonsmoking asthmatics.

### 4.1. Biomarkers of OS

#### Case-Control Studies

Asthmatic current smokers showed increased serum levels of malondialdehyde (MDA) and decreased levels of the ferric-reducing ability of plasma (FRAP) [37]. Higher MDA levels in exhaled breath condensate (EBC) have been reported in active smoking asthmatics than in their ex-smoking and nonsmoking counterparts [38]. The levels of protein carbonyls and peroxynitrite in plasma were reported to be higher in current smoking asthmatics than in their ex-smoking and nonsmoking counterparts [39]. Smoking asthmatics with a lower FEV_1_ have higher erythrocyte antioxidant enzyme activity, including superoxide dismutase (SOD) and disulfide/oxidized glutathione (GSH) activity, than nonsmoking patients with asthma and healthy controls. Increased SOD and GSH in smoking asthmatics may not protect airway epithelial cells against the harmful effects of free radicals. SOD and GSH activities were found to be higher in nonsmoking asthmatics than healthy controls [40]. Nonsmoking asthmatics demonstrated increased levels of nitrite (NO_2_^−^), protein carbonyls, lipid peroxide, SOD activity, and decreased protein sulfhydrils and glutathione peroxidase (GPx) activity in leukocytes and red blood cells [41]. High sputum GSH and NO_2_^−^ levels were reported in nonsmokers with stable and acute asthma [42].

Overall findings suggest that the oxidant/antioxidant imbalance derived by CS is likely to exist in smoking and nonsmoking asthmatics. OS biomarkers are increased in current and nonsmokers, but the increase in the enzymatic antioxidants in smokers may be insufficient to protect bronchial/airway epithelial cells against oxidative damage. Table 2 shows the OS biomarkers in smoking and nonsmoking asthmatics.

### 4.2. Biomarkers of Inflammation

#### 4.2.1. Case-Control Studies

Fractional exhaled nitric oxide (FeNO) was reported in lower levels in smoking asthmatics than nonsmoking asthmatics and healthy controls. This reduction is accompanied by increased numbers of sputum eosinophils [43]. Smoking asthmatics had lower FeNO levels than their nonsmoking counterparts, but this decrease does not appear to reflect improvement of asthma control [44]. Low levels of FeNO were observed in current/ex-smokers with severe asthma compared to nonsmokers with mild-moderate asthma. High FeNO levels and blood eosinophil count provide a moderate prediction of type 2 high status in severe asthmatic nonsmokers [45]. Increased levels of FeNO, eosinophils, and neutrophils have been observed in the airways of current nonsmoking asthmatics [46]. Compared to active smokers with asthma, nonsmokers with asthma had higher FeNO levels [47].

Eotaxin-1 in EBC was associated with blood eosinophil count, FeNO value, and serum ECP in nonsmoking asthmatics [48]. Eotaxin was found at higher levels in the sputum of smokers than nonsmokers with asthma. Sputum and serum IL-5 levels were found to be higher in nonsmoking asthmatics than in smoking asthmatics and healthy controls. High BALF eotaxin-1 was associated with increased BALF eosinophil and neutrophil counts and percentages [49]. Comparatively greater levels of sputum IL-1β, IL-5, and Interleukin 18 receptor 1 (IL-18R1) have been reported in current/ex-smoking severe asthmatics compared to healthy controls. Bronchoscopy and Bronchoalveolar Lavavge Fluid (BALF) levels of eotaxin-1 were observed to be high in nonsmoking asthmatics. Higher sputum levels of IL-4, IL-5, IL-1β, Interleukin 1 receptor-like 1 (IL-1RL1), Interleukin 1 receptor, type I (IL-1R1), IL-1R2, IL-18R1, and NLRP3 were detected in nonsmoking severe asthmatics compared to mild-moderate asthmatics and healthy controls [50].

Higher sputum eosinophils, eosinophilic cationic protein (ECP), neutrophils, and IL-8 levels were observed in asthmatic smokers compared to healthy nonsmokers, which were associated with FEV_1_ and neutrophil count. Compared to healthy nonsmokers, nonsmoking asthmatics demonstrated higher sputum ECP and eosinophil levels [51]. Serum periostin has been observed in higher levels in nonsmokers than smokers with asthma [52]. High serum periostin, TNFα, IL-4, IL-5, and the chitinase-like protein YKL-40 levels, as well as low serum IL-37 levels, were associated with exacerbated asthma in nonsmokers [53]. Asthmatic smokers demonstrated increased sputum levels of neutrophils, and decreased levels of eosinophils compared to asthmatic nonsmokers. Both asthmatic smokers and nonsmokers showed increased sputum levels of eosinophils compared to healthy smokers. High IL-18 levels in the sputum of nonsmoking asthmatics were associated with FEV_1_ decline [54]. Current and ex-smokers with asthma have higher frequencies of sputum type 3 innate lymphoid cells (ILC3), which has been identified as a biomarker of airway eosinophilic inflammation, and peripheral blood CD45RO-expressing memory-like ILC3s compared with nonsmokers counterparts. ILC3 was associated with M1 alveolar macrophage and circulating neutrophil counts [55]. High levels of peripheral blood ILC2, FeNO, blood eosinophils, and serum IgE were associated with sputum eosinophil counts in eosinophilic asthmatic nonsmokers compared to healthy controls [56]. Higher serum high-sensitivity C-reactive protein (hs-CRP) levels were reported in nonsmokers with mild-to-moderate asthma than healthy controls and were associated with sputum neutrophils/eosinophils and impaired FEV_1_ [57]. Nonsmoking asthmatics showed higher levels of hs-CRP and blood eosinophils compared to nonsmoking healthy controls [58]. A number of inflammation biomarkers, including hs-CRP, serum total IgE, and blood/sputum eosinophils and neutrophils have been identified in higher proportions in smokers and nonsmokers with severe asthma compared to healthy nonsmokers [59]. Higher levels of Matrix metallopeptidase (MMP-12), C-X-C motif chemokine ligand-8 (CXCL8), neutrophil elastase, azurocidin 1 (AZU-1), and pro-platelet basic protein (PPBP) were observed in the sputum of ex-smoking asthmatics, which are linked to neutrophilic inflammation. Nonsmokers with asthma have significantly elevated sputum and blood eosinophil counts [60].

#### 4.2.2. Cross-Sectional Studies

FeNO levels were reported to be high in ex-smoking and nonsmoking asthmatics. Low FeNO levels were associated with increased nitric oxide synthase (NOS2) mRNA levels in current smokers, but not in ex-smokers/nonsmokers. Current and ex-smoking asthmatics exhibit higher NADPH oxidase 2 (NOX2) mRNA levels [61]. Current smokers with severe asthma have lower FeNO value, sputum eosinophils/neutrophils, and serum-specific IgE levels than nonsmokers. Ex-smokers compared with nonsmokers have higher sputum neutrophils, blood eosinophils, and lower serum-specific IgE levels [62]. FeNO levels have been observed higher in nonsmokers than current and ex-smokers with asthma. Current and ex-smokers compared with nonsmokers had higher number of blood eosinophils [63]. FeNO values were higher in smokers and nonsmokers with uncontrolled asthma treated and/or treated with ICS than those with partly/well-controlled asthma [64]. Current smoking was associated with small airway obstruction in asthma. Interestingly, increased levels of serum IgE were associated with reduced risk of small airway obstruction in nonsmoking asthmatics compared to current and ex-smoking counterparts [65].

#### 4.2.3. Cohort Studies

Higher FeNO levels have been observed in nonsmokers than smokers with asthma, which are associated with FEV_1_ and FEV_1_/FVC decline over 20-year follow-up [66]. In asthmatic patients with persistent obstruction where nonsmokers represented the vast majority, high sputum periostin levels were associated with FEV_1_ decline and high sputum eosinophil counts, resulting in increased FeNO value, blood eosinophil counts and transforming growth factor beta 1 (TGF-β1) over 2-year follow-up [67].

It can be suggested that current smoking, ex-smoking, and nonsmoking asthmatics exhibit higher levels of inflammation biomarkers, which have the potential to increase the risk. Table 3 shows the OS and inflammatory biomarkers in nonsmoking asthmatics.

## 5. Potential Effects of Antioxidant on CS-Induced Asthma Biomarkers

### 5.1. Antioxidant Vitamins

#### 5.1.1. Vitamin A

Vitamin A (VA) derived from dietary animal-source foods has an active metabolite retinoic acid (RA), which binds retinoic acid receptors (RARs) and retinoid X receptors (RXRs) with high affinity, resulting in a regulation of ASM cell proliferation in asthma [68]. Low RA levels in ASM cells increases the severity of asthma [69]. Human ASM cells treated with RARγ-specific agonist and all-*trans* RA (ATRA) lead to the inhibition of platelet-derived growth factor (PDGF)-induced activator protein-1 (AP-1) regulated genes, including MMP8 and MMP9 [69]. TGF-β increases the expression of ATRA and 9-*cis* RA in the ASM cells of patients with severe asthma compared with those with mild-to-moderate asthma, which results in upregulation of the mRNA of β1-integrin, MMP-9, and hepatocyte growth factor receptor (HGF-R). Treatment with anti-TGF-β1 monoclonal antibody in the presence of ATRA/9-*cis* RA reduces the levels of MMP-9 mRNA in ASM cells. This concludes that TGF-β increases ASM cell inflammation in response to exaggerated RA receptor expression, which may lead to airway epithelial repair defects in severe asthma [70]. Administration of ATRA suppresses PDGF-induced ASM cell migration via RAR-RXR heterodimer activation and Serine-threonine kinase/Phosphatidylinositol-3 kinase (Akt/PI3K) signaling pathway inhibition [71]. Treatment with RA improves barrier strength of HBECs by reducing TNF-α and IL-6-induced airway barrier leaks and occluded din/claudin-4 [72]. ATRA treatment inhibits airway inflammation by suppressing Th2 and Th17-related cytokines (IL-4, IL-5, IL-17A), neutrophils, eosinophils, macrophages, and lymphocytes counts [73]. Administration of ATRA and 9-*cis* RA suppresses IL-4-induced eotaxin mRNA expression in HBECs [74]. 9-*cis*-RA treatment results in reversing RAR-beta (RAR-β) expression loss in the HBECs of ex-smokers, suggesting that RA may be considered as a potential agent against asthma risk in smokers [75]. Nonsmoker patients with lung emphysema treated with ATRA resulted in improvement in lung function and reduction of airway inflammation through the inhibition of TNF-α and IL-3 plasma levels [76]. Overall findings suggest that VA exerts anti-inflammatory effects on ASM/HBECs cells.

#### 5.1.2. Carotenoids

β-carotene, also termed provitamin A/non-polar carotenoid, and other non-provitamin A/polar carotenoids (e.g., lycopene, lutein, zeaxanthin) are natural pigments, present primarily in fruits and vegetables, which have been shown to exert anti-inflammatory/OS agents for several diseases [77], including asthma [78]. It has been shown that supplementation of HBECs “BEAS-2B” with β-carotene does not promote membrane LP/lactate dehydrogenase (LDH) leakage and α-tocopherol/GSH depletion caused by gas phase smoke [79]. β-carotene exerts a protective effect in HBECs treated with CS-induced lung carcinogen benzo[a]pyrene (BaP) through increasing RAR-β expression [80].

Lycopene exerts a therapeutic effect against asthma by reducing eosinophilic infiltrates and Th2-mediated cytokines IL-4 and IL-5 production in the airways [81]. Treatment with apo-10′-lycopenoic acid, an active metabolite of lycopene, increases accumulation of nuclear factor-E2 related factor 2 (Nrf2)-mediated heme-oxygenase-1 (HO-1) activation and intracellular GSH levels and decreases intracellular ROS levels and hydrogen peroxide (H_2_O_2_)-induced LDH production in BEAS-2B [82]. Treatment of BEAS-2B with β-cryptoxanthin (BCX) reduces inflammation, as indicated by increased sirtuin1 (SIRT1) protein levels and inhibited lipopolysaccharide (LPS)-induced TNF-α, MMP2/9, IL-6, and monocyte chemoattractant protein-1 (MCP-1) mRNA levels [83]. BCX supplementation of ferrets led to inhibited CS-induced NF-kB, AP-1, and TNFα expression in the lungs [84]. These findings suggest that lycopene and BCX may protect HBECs against CS-induced inflammation and/or OS biomarkers.

#### 5.1.3. Vitamin C and E

Epidemiological studies regarding the role of antioxidant VC and VE in the treatment of asthma have demonstrated inconsistent findings [85]. The ascorbic acid-supplemented diet has been shown to reduce the bronchoconstrictive responses in asthmatic patients, as demonstrated by decreasing post-exercise FeNO, FEV_1_, and urinary 9α, 11β-PGF2 levels [86]. Administration of VC to ovalbumin (OVA)-sensitized and challenged asthmatic mice attenuates airway inflammation by reducing eosinophilic infiltration into BALF [87].

VE and curcumin treatment reduces BaP-induced ROS levels by downregulating poly[ADP-ribose] polymerase 1 (PARP-1) and protein 53 (p53) activity in BEAS-2B [88]. Treatment with natural-source d-α-tocopheryl acetate increases plasma levels of α-tocopherol isoform of VE in atopic asthmatics, resulting in reduced BAL levels of IL-3 and IL-4 [89]. In allergic asthmatic adults, γ-tocopherol treatment led to suppression of LPS-induced TNFα, IL-6, and IL-1β production from peripheral blood monocytes [90]. In human and mice models, supplementation with γ-tocopherol reduced LPS-induced sputum percentages of neutrophils and eosinophils [91]. γ-tocopherol supplementation reduces sputum mucins, neutrophils, and eosinophils in mild asthmatics [92]. VE-supplemented diet results in decreased IL-4 and IL-5 levels in the murine lungs [93]. Treatment with VE attenuates AHR through reducing OS and inflammatory biomarkers. VE decreases LPS-induced IL-5, IL-13 levels, H2O2-mediated ROS production, eosinophils, neutrophils, and restores the serum activity of GSH and SOD [94]. Administration of VE reduces asthma by decreasing IL-4 levels, ROS production, serum IgE levels, and increasing GSH levels [95]. VE treatment attenuates allergic asthma by reducing levels of peroxynitrite, NO_2_^−^, IgE, eotaxin, IgE, TGF-β1, IL-4, IL-5, and IL-13 [96]. Administration of supplemental α-and γ-tocopherol resulted in reduced BALF IL-5, IL-12, and IL-13 levels [97]. This suggests that VC may reduce airway inflammation, while VE may have protective effects against both OS and inflammatory biomarkers.

#### 5.1.4. Vitamin D

Evidence from in vitro and in vivo studies has supported the protective role of VD against asthma, by which VD supplementation reduces airway inflammation and improves lung function in asthmatic patients [98]. VD treatment has been shown to decrease IL-6 and CXCL8 levels in cultured HBECs from asthmatic donors [99]. In asthmatic patients, supplementation with VD increases serum anti-inflammatory IL-10, and decreases serum levels of IgE, eosinophil, IL-5, IL-9, and IL-13 [100]. Supplementation with VD reduces asthmatic airway inflammation, as evidenced by decreased levels of IL-4 in BALF and NO_2_^−^ in serum and BALF [101]. VD treatment decreases the index of airway collagen deposition, mucus reserve, and increases autophagy-related protein expression levels of hypoxia-inducible factor 1 alpha (HIF-1α) and neurogenic locus notch homolog protein 1 (Notch1), resulting in reduced airway inflammation associated with IL-6 and IL-17 cytokines [102]. Supplementation of VD reduces AHR and IgE levels in BALF and serum in asthmatic mice [103]. Administration of VD to VD-deficient mice with asthma reduces BALF levels of neutrophil, eosinophil, IL-5, and IL-13 [104]. It has been demonstrated that 1,25(OH)(2)D(3) supplementation reduces serum OVA-specific IgE levels, accompanied with increased serum levels of IL-10 via inhibition of NF-kB signaling pathway [105]. VD supplementation reduces BALF eosinophil numbers, BALF IL-6, IL-17, TNF-α levels, and increase BALF IL-10 levels [106]. VD was found to reduce serum levels of IL-6, IL-1β, TNF-α, and increase serum levels of IL-10 through downregulating high mobility group box 1 proteins (HMGB1)/TLR4/NF-κB signaling pathway [107]. Overall findings suggest that VD may have anti-inflammatory/anti-oxidants effects on HBECs due to its ability to reduce inflammatory biomarkers, which may therefore be involved in CS-induced asthma treatment.

### 5.2. Antioxidant Minerals

#### 5.2.1. Iron

Iron (Fe) is a critical mineral implicated in free radical production, which has a detrimental effect on asthma, as evident by increasing plasma Fe levels in HBECs, which result in a significant increase in OS and LP [108,109]. IL-6 was shown to enhance ferroptosis in HBECs, identified as regulated cell death, by disrupting iron homeostasis and increasing ROS and MDA-dependent LP [110]. In mice sensitized to OVA, high expression of HO-1, an enzyme responsible for degrading heme into free iron, was found to be associated with airway inflammation via increased levels of IL-5, IL-13, and eosinophilia in the lung tissue [111].

High serum levels of saturation of transferrin and ferritin, as indices of Fe homeostasis, were associated with airway obstruction in smokers and nonsmokers with the lowest FEV_1_/FVC ratio [112]. Exposure to tobacco smoke condensate alters iron homeostasis in human respiratory epithelial cells by increasing serum Fe and ferritin accumulation in the lungs of smokers [113]. This suggests that Fe is associated with increased asthma risk and should not be recommended for smoking asthmatics.

#### 5.2.2. Zinc, Selenium, and Copper

Evidence from in vivo and in vitro studies suggests that zinc (Zn), Se, and copper (Cu) play a significant role in reducing asthma and protecting airway epithelial cells against OS and inflammatory biomarkers [114]. Treatment of airway epithelial (HEp-2) cells with toxic copper oxide nanoparticles (CuONPs) results in induced OS by increasing ROS and 8-isoprostane production [115]. CuONPs increase AHR and the production of ROS and pro-inflammatory cytokines via activating of MAPK signaling in OVA-induced asthmatic mice [116]. Cu and Zn are key components of SOD, which results in a reduction of OS. The plasma levels of Se, Cu, Zn, and a cytosolic antioxidant enzyme, copper–zinc-superoxide dismutase (CuZnSOD), were reported to be lower in asthmatics than in healthy controls [117,118,119]. Se was found to be associated with decreased levels of OS biomarker plasma thiobarbituric acid-reactive substances (TBARS), hs-CRP levels and CD4/CD8 lymphocyte ratios [118]. An experimental study has demonstrated that Zn chelator N,N,N′,N′-tetrakis(2-pyridylmethyl)ethylenediamine inhibits TNFα-induced eotaxin mRNA expression in BEAS-2B cells [120]. Zn supplementation reduced BALF eosinophils and attenuated airway inflammation-induced solute carrier family 39 members 1 and 14 (ZIP1, ZIP14) [121]. Administration of Zn to mice with OVA-induced allergic asthma led to reduced monocytes, neutrophils, and eosinophils in BALF, MCP-1, and eotaxin protein production [122]. This suggests that Zn may have potential antioxidant effects against inflammation biomarkers.

The potential effect of antioxidant vitamins and minerals on CS-induced asthma biomarkers was shown in Table 4.

## 6. Concluding Remarks

CS is associated with biomarkers of OS and systemic inflammation in HBECs. Literature has shown that OS and inflammation have a significant role in the pathogenesis of asthma in current smokers, ex-smokers, and nonsmokers. There were significant differences in asthma biomarkers between smokers and nonsmokers. OS biomarkers MDA, FRAP, protein carbonyls, and peroxynitrite, and inflammation biomarkers ILC2/3, MMP-12, CXCL8, neutrophil elastase, AZU-1, and PPBP were found to associate with asthma in smokers, but not in nonsmokers. However, OS biomarkers NO_2_^−^, lipid peroxide, protein sulfhydrils, and inflammatory biomarkers TNFα, IL-4, IL-37, IL-1β, IL-1RL1, IL-1R1, NLRP3, and TGF-β1 showed positive association with asthma in nonsmokers, but not in smokers. Current smokers with asthma have higher levels of OS biomarkers than nonsmokers and are thus at a heightened state of OS. The activity of the enzymatic antioxidant defense in smoking asthmatics may not adequately protect the HBECs against oxidative damage.

Evidence from a few studies suggests that dietary VC and carotenoid intake are associated with reduced asthma risk in smokers and/or nonsmokers. Supplementing VE and Se had no effects on improving lung function in smoking asthmatics.

Several in vivo and in vitro studies have demonstrated the protective effects of antioxidant vitamin and mineral against asthma biomarkers. VA, VC, BCX, and Zn might protect HBECs against inflammatory biomarkers, while VE, VD, and lycopene might provide protection against both OS and inflammatory biomarkers. Fe has adverse effects on HBECs and should be avoided for smoking and nonsmoking asthmatics.

The potential effects of antioxidant on CS-induced asthma biomarkers in smoking and nonsmoking asthmatics are difficult to determine, given a limited number of human studies. VA and carotenoids may trigger a protective effect against asthma in smokers and/or nonsmokers. However, VC, VE, VD, and Zn may have protective potential against asthma biomarkers. Such effects lead to the conclusion that these antioxidants might have beneficial effects in reducing asthma in smokers and nonsmokers, given that smoking and nonsmoking asthmatics are susceptible to CS-induced OS and inflammatory biomarkers.

The mechanisms by which antioxidant vitamins and minerals might be effective in protecting HBECs against asthma biomarkers in smokers and nonsmokers have not been fully elucidated. Human studies on the exact mechanisms (signaling pathways) linking the antioxidant intake to asthma in smokers and nonsmokers have not yet been confirmed. Few signaling pathways might be involved, as demonstrated by in vivo models (e.g., TLR4/NF-κB signaling pathway in VD). Further human studies are needed to explore the mechanisms by which antioxidants might be effective in protecting HBECs against asthma biomarkers in smokers and nonsmokers.

More studies on smokers and nonsmokers are needed to determine the associations between antioxidant intake from both diet and supplements and asthma biomarkers. Studies included in this review did not determine whether nonsmokers with asthma are affected by SHS exposure. Thus, further studies are required to examine whether antioxidant intake in nonsmoking asthmatics could protect against CS-induced asthma biomarkers.

## Figures and Tables

**Table 1:** Antioxidants and asthma risk in relation to smoking status.

Design	Study Population	Antioxidants	Main Findings	Ref.
Cross-sectional	Total subjects = 2112 ^1^2th grade US students	VC, VE (diet)	Low dietary VC intake (<110 mg/day) was associated with FEV_1_ decline and respiratory symptoms in smokers with asthma	[32]
	Smokers = 515		VE intake was not associated with asthma	
Cross-sectional	Total subjects = 13,039 US adults (20–80 yrs)	Total carotenoids (diet and supplement)	High intake of carotenoids (≥165.59 μg/kg/day) was associated with reduced asthma risk in nonsmokers (OR = 0.63, 95% CI = 0.42 to 0.93), current smokers (OR = 0.54, 95% CI = 0.36 to 0.83), and ex-smokers (OR = 0.64, 95% CI = 0.42 to 0.97)	[33]
	Current asthma = 1784; non-current asthma = 11,255			
	Nonsmokers= 7106; current smokers= 3304; ex-smokers= 2624			
RDBPC	Total subjects = 72 UK nonsmoking asthmatics (18–60 yrs)	VE (supplement)	VE had no beneficial effects on asthma	[34]
		500 mg VE capsules (D-α-tocopherol) in soya bean oil or matched placebo (capsules, gelatine base) for 6 weeks		
RDBPC	Total subjects = 197 UK smoking and nonsmoking asthmatics (18–54 yrs)	Se (supplement)	Plasma Se was increased by 48% in the Se group. However, no significant improvement in QoL score was observed in the Se group compared with placebo	[35]
		100 μg/day high-Se yeast preparation or matched placebo (yeast only) for 24 weeks		

Abbreviations: RDBPC, randomized double blind placebo control; VC, vitamin C; VE, vitamin E; Se, selenium; QoL, quality of life; OR, odds ratio.

**Table 2:** OS biomarkers in smoking and nonsmoking asthmatics.

Design	Study Population	OS Biomarkers	Ref.
Case-control study	Total subjects = 210 Indian (13–80 yrs) Smokers/nonsmokers (asthmatics = 19/101; healthy controls = 29/61)	Asthmatic smokers = MDA ↑, FRAP ↓	[37]
Case-control study	Total subjects = 194 Italian patients with different pulmonary diseases (average 45.8 yrs)	Asthmatic current smokers = MDA ↑	[38]
	Asthmatics (current and ex-smokers) = 64; healthy controls (nonsmokers) = 14		
Case-control study	Total subjects = 329 Tunisian adults (average 43.6 yrs)	Asthmatic current smokers = Protein carbonyls, peroxynitrite ↑	[39]
	Asthmatic current smokers/healthy controls = 14/73; Asthmatic ex- smokers/healthy controls = 17/13		
	Asthmatic nonsmokers/healthy controls = 120/92		
Case-control study	Total subjects = 266 Chinese adults (39–47 yrs)	Asthmatic smokers and nonsmokers = SOD, GSH ↑	[40]
	Asthmatic smokers/nonsmokers = 25/106; healthy controls (nonsmokers) = 135		
Case-control study	Total subjects = 61 Indian (15–40 yrs) Asthmatic nonsmokers/healthy controls= 38/23	Asthmatic nonsmokers = SOD, NO_2_^−^, protein carbonyls, lipid peroxide ↑	[41]
		GPx, protein sulfhydrils ↓	
Case-control study	Total subjects = 32 Turkish adults (average 41 yrs)	Asthmatic nonsmokers = GSH, NO_2_^−^ ↑	[42]
	Stable asthmatic nonsmokers = 11; Severe asthmatic nonsmokers = 10; Healthy nonsmokers = 11		

(↓) decrease, (↑) increase.

**Table 3:** Inflammatory biomarkers in smoking and nonsmoking asthmatics.

Design	Study Population	Inflammation Biomarkers	Ref.
Case-control study	Total subjects = 143 Greek adults (average 48.7 yrs)	Asthmatic smokers = FeNo ↓, eosinophils ↑	[43]
	Asthmatic smokers/nonsmokers = 40/43		
	Healthy smokers/nonsmokers = 30/30		
Case-control study	Total subjects = 470 Belgium adults (average 41 yrs)	Asthmatic smokers = FeNo ↓	[44]
	Asthmatic smokers/nonsmokers = 59/411		
Case-control study	Total subjects = 147 European adults (average 46.8 yrs)	Asthmatic smokers = FeNo ↓	[45]
	Asthmatic smokers = 18; Severe Asthmatic nonsmokers = 49; Mild-moderate asthmatic nonsmokers = 36; healthy nonsmoker = 44	Asthmatic nonsmokers = FeNo, eosinophil ↑	
Case-control study	Total subjects = 1230 Italian adults (20–65 yrs)	Asthmatic nonsmokers = FeNo, eosinophils, neutrophils ↑	[46]
	Current/past asthmatic nonsmokers = 404/185; Current and past chronic bronchitis smokers = 92; healthy controls = 549		
Case-control study	Total subjects = 282 Danish (14–44 yrs)	Asthmatic nonsmokers = FeNo, ↑	[47]
	Asthmatic current/ex-smokers = 112/62; Asthmatic nonsmokers = 108		
Case-control study	Total subjects = 58 Polish adults (25–45 yrs)	Asthmatic nonsmokers = FeNo, eotaxin, eosinophil ↑	[48]
	Asthmatic nonsmokers/Healthy controls = 46/12		
Case-control study	Total subjects = 68 Lithuanian adults (average 55.2 yrs)	Asthmatic smokers = Eotaxin, neutrophils, eosinophils ↑	[49]
	Asthmatic smokers/nonsmokers = 19/26; Healthy smokers and non-smokers = 23	Asthmatic nonsmokers = Eotaxin, neutrophils, eosinophils, IL-5 ↑	
Case-control study	Total subjects = 86 UK adults (average 50 yrs)	Asthmatic smokers = IL-1β, IL-5, IL-18R1 ↑	[50]
	Severe asthmatic smokers/nonsmokers = 21/37; mild-moderate asthmatics = 15; healthy controls = 13	Asthmatic nonsmokers = Eotaxin, IL-4, IL-5, IL-1β, IL-1RL1, IL-1R1, NLRP3 ↑	
Case-control study	Total subjects = 97 UK adults (average 37 yrs)	Asthmatic smokers = eosinophils, ECP, neutrophils, IL-8 ↑	[51]
	Asthmatic smokers/nonsmokers = 31/36; Nonasthmatic smokers/nonsmokers = 15/15	Asthmatic nonsmokers = eosinophils, ECP ↑	
Case-control study	Total subjects = 152 UK adults (18–75 yrs)	Asthmatic smokers = Periostin ↓	[52]
	Asthmatic smokers/nonsmokers = 56/51; Healthy smokers/nonsmokers = 20/25	Asthmatic nonsmokers = Periostin ↑	
Case-control study	Total subjects = 89 Turkish adults (25–65 yrs)	Asthmatic nonsmokers = Periostin, TNFα, IL-4, IL-5, YKL-40 ↑, IL-37 ↓	[53]
	Asthmatic nonsmokers/healthy controls = 59/30		
Case-control study	Total subjects = 79 Greek adults (average 46 yrs)	Asthmatic smokers = eosinophils, neutrophils ↑, IL-18 ↓	[54]
	Asthmatic smokers/nonsmokers = 24/22; Healthy smokers/nonsmokers = 16/17	Asthmatic nonsmokers = eosinophils, neutrophils, IL-18 ↑	
Case-control study	Total subjects = 115 Korean adults (average 55 yrs)	Asthmatic smokers = ILC3, eosinophils, neutrophils ↑	[55]
	Asthmatic smokers/nonsmokers = 58/33; Healthy smokers/nonsmokers = 11/13		
Case-control study	Total subjects = 168 Chinese adults (average 36 yrs)	Eosinophilic asthmatic nonsmokers = ILC2, IgE, eosinophils, FeNO ↑	[56]
	Eosinophilic asthmatic/non asthmatic nonsmokers = 62/64; Healthy controls = 42		
Case-control study	Total subjects = 85 Japanese adults (20–60 yrs)	Asthmatic nonsmokers = hs-CRP, eosinophils, neutrophils ↑	[57]
	Asthmatic nonsmokers/healthy controls = 45/40		
Case-control study	Total subjects = 98 Iranian adults (average 35 yrs)	Asthmatic nonsmokers = hs-CRP, eosinophils ↑	[58]
	Asthmatic nonsmokers receiving/not receiving inhaled fluticasone (500 µg/day) = 31/30; Healthy controls = 37		
Case-control study	Total subjects = 525 European adults (36–55 yrs)	Asthmatic smokers and nonsmokers = hs-CRP, eosinophils, neutrophils, IgE ↑	[59]
	Smokers or ex-smokers with severe asthma = 95; Nonsmokers with severe asthma = 263; Nonsmokers with mild-moderate asthma = 76; healthy nonsmoker = 91		
Case-control study	Total subjects = 88 European adults (39–50 yrs)	Asthmatic ex-smokers = MMP-12, CXCL8, neutrophil elastase, AZU-1, PPBP ↑	[60]
	Asthmatic current smokers = 11; Asthmatic ex-smokers = 22; Asthmatic nonsmoker s = 37; Healthy nonsmoker = 18	Asthmatic nonsmokers = eosinophil ↑	
Cross-sectional study	Total subjects = 324 European adults with severe asthma (average 52.5 yrs)	Asthmatic current smokers = FeNo ↓, NOS_2_, NOx_2_ ↑	[61]
	Current smokers = 42; Ex-smokers = 112; Nonsmokers = 260	Asthmatic ex-smokers = FeNo, NOX_2_ ↑	
		Asthmatic nonsmokers = FeNo ↑	
Cross-sectional study	Total subjects = 740 UK patients with severe asthma (6–43 yrs)	Asthmatic current smokers = FeNO, blood eosinophils, sputum eosinophils	[62]
	Current smokers = 69; Ex-smokers = 210; Nonsmokers = 461	sputum neutrophils, IgE ↓	
		Asthmatic ex-smokers = FeNO, sputum neutrophils, blood eosinophils ↑, IgE ↓	
		Asthmatic nonsmokers = FeNO, IgE, sputum neutrophils, sputum eosinophils, blood eosinophils ↑	
Cross-sectional study	Total subjects = 1578 French patients with asthma (40–64 yrs)	Asthmatic current and ex- smokers = FeNO ↓, blood eosinophils ↑	[63]
	Current smokers = 294; Ex-smokers = 473; Nonsmokers = 812	Asthmatic nonsmokers = FeNO ↑, blood eosinophils ↓	
Cross-sectional study	Total subjects = 274 Greek patients with asthma (average 50 yrs)	Asthmatic smokers and nonsmokers = FeNO ↑	[64]
	Inhaled corticosteroid (ICS)-treated smokers = 50; ICS-untreated smokers = 32; ICS-treated nonsmokers = 144; ICS-untreated nonsmokers = 48		
Cross-sectional study	Total subjects = 478 Chinese patients with asthma (average 45 yrs)	Asthmatic nonsmokers = IgE ↑	[65]
	Obstructive group (current smokers = 70; ex-smokers = 44; nonsmokers = 271), Normal group (current smokers = 9; ex-smokers = 6; nonsmokers = 78)		
Prospective cohort study	Total subjects = 4257 European and Australian adults (average 54 yrs)	Asthmatic nonsmokers = FeNO ↑	[66]
	Current asthma (smokers = 97; nonsmokers = 554)		
	Non-asthma (smokers = 651; nonsmokers = 2955)		
Prospective cohort study	Total subjects = 45 Italian adults with severe asthma (average 60 yrs)	Asthmatic nonsmokers = FeNO, periostin, eosinophil, TGF-β1 ↑	[67]
	Nonsmokers = 42; ex-smokers = 3		

(↓) decrease, (↑) increase.

**Table 4:** Potential influence of antioxidant vitamins and minerals on CS-induced asthma biomarkers.

Study Design	Antioxidant Vitamins/Minerals	Concentration/Supplement Intake	OS Biomarkers	Inflammation Biomarkers	Ref.
In vitro	VA (RARγ, ATRA)	1–1000 μM	-	MMPs (MMP8, MMP9) ↓	[69]
In vitro	VA (ATRA, 9-*cis* RA)	1 mM in the presence of 0.2 mg/mL neutralizing anti-TGF-β1	-	MMP9 ↓	[70]
In vitro	VA (RA)	5, 15, 50 μM	-	TNFα, IL-6 ↓	[72]
In vivo	VA (ATRA)	400 μg/mL	-	IL-4, IL-5, IL-17A, neutrophils, eosinophils ↓	[73]
In vitro	VA (ATRA, 9-*cis* RA)	10^−6^–10^−10^ M	-	IL-4, eotaxin ↓	[74]
In vivo	Lycopene	0.16 mg (equivalent to 8 mg/kg per day) plus 0.05 mg VE and 0.006 mg β-carotene	-	IL-4, IL-5, eosinophils ↓	[81]
In vitro	Lycopene	3, 5 10 μM	GSH ↑, ROS, H_2_O_2_ ↓	-	[82]
In vivo/vivo	BCX	1–4 μM (in vitro) 30−43 nmol BCX/g liver (in vivo)	-	Neutrophils, TNFα, IL-6, MMPs (MMP2, MMP9) ↓	[83]
RCT	VC	1500 mg/day for 2 weeks, or a placebo	-	FeNO ↓	[86]
In vivo	VC	130 mg VC/kg bw/day	-	Eosinophils ↓	[87]
Non-randomized trial in vivo	VE	1500 IU/day for 16 weeks	-	IL-3, IL-4 ↓	[89]
ex-vivo	VE	1200 mg/day for 8 days	-	TNFα, IL-1β, IL-6 ↓	[90]
RDBPC	VE	1200 mg/day for 14 days, or a placebo	-	Eosinophils, neutrophils ↓	[92]
In vivo	VE	50 mg VE/kg bw/day	H_2_O_2_, ROS ↓, GSH, SOD ↑	IL-5, IL-13, eosinophils, neutrophils ↓,	[94]
In vivo	VE	0.2 and 2.0 mg VE/kg bw/day	ROS ↓, GSH ↑	IL-4, IgE ↓	[95]
In vivo	VE	5, 10, 15, and 20 IU VE/kg bw/day	NO_2_^−^, peroxynitrite ↓	IL-4, IL-5, IL-13, eotaxin, IgE, TGF-β1 ↓	[96]
In vivo	VE	0.2 or 2 mg VE/kg bw/day	-	IL-5, IL-12, IL-13 ↓	[97]
In vitro	VD	100 μM	-	IL-6, CXCL8 ↓	[99]
RCT	VD	0.25 μg/day calcitriol for 6 months, or a placebo	-	IL-5, IL-9, IL-13, IgE, eosinophils ↓, IL-10 ↑	[100]
In vivo	VD	50 μg/kg VD/kg bw/day	NO_2_^−^ ↓	IL-4 ↓	[101]
In vivo	VD	100, 500, or 1000 IU VD/kg bw/day	-	IL-6, IL-17 ↓	[102]
In vivo	VD	10,000 IU VD/kg bw/day	-	IgE ↓	[103]
In vivo	VD	2280 IU VD/kg bw/day	-	IL-5, IL-13, eosinophils, neutrophils ↓	[104]
In vivo	VD	1 mg VD/kg bw/day	-	IgE ↓, IL-10 ↑	[105]
In vivo	VD	10,000 IU VD/kg bw/day	-	IL-6, IL-17, TNFα, eosinophils ↓, IL-10 ↑	[106]
In vivo	VD	1 μg/mL/20 g VD/kg bw/day	-	IL-6, IL-1β, TNFα, ↓, IL-10 ↑	[107]
In vitro	Fe	3.3 M plus ferroptosis inhibitor Fer-1 (0.1 μM)	MDA, ROS ↑, GSH ↓	IL-6 ↑	[110]
In vitro	Zn	2 μM	-	TNFα, eotaxin ↓	[120]
In vivo	Zn	0, 54, or 100 μg Zn/kg bw/day	-	Eosinophils ↓	[121]
In vivo	Zn	95 mg Zn/kg bw/day	-	Eosinophils, neutrophils, eotaxin ↓	[122]

Abbreviations: RDBPC, randomized double blind placebo control; RCT, randomized controlled trial; VA, vitamin A; ATRA, All-*trans* RA; RA, retinoic acid; RAR, retinoic acid receptors; BCX, β-cryptoxanthin; VC, vitamin C; VE, vitamin E; VD, vitamin D; Fe, iron; Zn, zinc. (↓) decrease, (↑) increase.

## Data Availability

Not applicable.

## Abbreviations

AHR	Airway hyperresponsiveness
Akt	Serine-threonine kinase
AP-1	Activator protein-1
ASM	Airway smooth muscle
ATRA	All-*trans* RA
AZU-1	Azurocidin 1
BALF	Bronchoalveolar Lavavge Fluid
BaP	Benzo[a]pyrene
BCX	β-cryptoxanthin
COPD	Chronic obstructive pulmonary disease
CS	Cigarette smoke
CSE	Cigarette smoke extract
Cu	Copper
CuONPs	Copper oxide nanoparticles
CuZnSOD	Zinc-superoxide dismutase
CXCL	C-X-C motif chemokine ligand
EBC	Exhaled breathe condensate
ECP	Eosinophilic cationic protein
ERK	Extracellular signal-regulated kinases
ETS	Environmental tobacco smoke
Fe	Iron
FeNO	Fractional exhaled nitric oxide
FEV_1_	Forced expiratory volume in 1 s
FRAP	Ferric reducing ability of plasma
FVC	Forced vital capacity
GPx	Glutathione peroxidase
GSH	Reduced glutathione
H_2_O_2_	Hydrogen peroxide
HBECs	Human bronchial epithelial cells
HDAC	Histone deacetylase
HGFR	Hepatocyte growth factor receptor
HIF-1α	Hypoxia-inducible factor 1 alpha
HMGB1	Mobility group box 1 protein
HO-1	Heme-oxygenase-1
hs-CRP	High-sensitivity C-reactive protein
ICS	Inhalation corticosteroid
IgE	Immunoglobulin E
IL	Interleukin
ILC	Innate lymphoid cell
IL-18R1	Interleukin 18 receptor 1
IL-1RL1	Interleukin 1 receptor-like 1
8-iso-PGF_2α_	_I_soprostane-8-iso prostaglandin F_2α_
JNK	c-Jun N-terminal kinase
LABA	Long-acting β2 adrenergic
LC	Lung cancer
LDH	Lactate dehydrogenase
LP	Lipid peroxidation
LPS	Lipopolysaccharide
MAPKs	Mitogen-activated protein kinases
MCP-1	Monocyte chemoattractant protein-1
MDA	Malondialdehyde
MMP	Matrix metallopeptidases
MPO	Myeloperoxidase
α7nAChR	α7 nicotinic acetylcholine receptor
NF-κB	Nuclear transcription factor-kappaB
NLRP	NLR Family CARD Domain Containing
NNK	Nitrosamine 4(methylnitrosamino)-1-(3–pyridyl)-1-butanone
NO_2_^−^	Nitrite
NOS	Nitric oxide synthase
Notch1	Neurogenic locus notch homolog protein 1
NOX2	NADPH oxidase 2
Nrf2	Nuclear factor-E2 related factor 2
OS	Oxidative stress
OVA	Ovalbumin
OXSR1	Oxidative stress responsive kinase 1
P53	Protein 53
PARP-1	Poly[ADP-ribose] polymerase 1
PDGF	Platelet-derived growth factor
PEFR	Peak expiratory flow rate
PGF2	Prostaglandin F2
PI3K	Phosphatidylinositol-3 kinase
PPBP	Pro-platelet basic protein
QoL	Quality of life
RA	Retinoic acid
RARs	Retinoic acid receptors
RDBPC	Randomized double blind placebo control
RXRs	Retinoid X receptors
RCTs	Randomised controlled trials
ROS	Reactive oxygen species
Se	Selenium
SHS	Secondhand smoke
SIRT1	Sirtuin1
SLC-39	Solute carrier family 39
SOD	Superoxide dismutase
TBARS	Thiobarbituric acid reactive substances
TGF-β1	Transforming growth factor beta 1
Th	T-helper
TLR	Toll-like receptors
TNF-α	Tumor necrosis factor α
TNFRSF11A	TNF receptor superfamily member 11a
VA	Vitamin A
VC	Vitamin C
VD	Vitamin D
VE	Vitamin E
Zn	Zinc

## References

[1] Gan H., Hou X., Zhu Z., Xue M., Zhang T., Huang Z., Cheng Z.J., Sun B. (2022). Smoking: A leading factor for the death of chronic respiratory diseases derived from Global Burden of Disease Study 2019. BMC Pulm. Med..

[2] Institute for Health Metrics and Evaluation (2017). Global Burden of Disease 2017. http://vizhub.healthdata.org/gbd-compare/#.

[3] Aoshiba K., Nagai A. (2004). Differences in airway remodeling between asthma and chronic obstructive pulmonary disease. Clin. Rev. Allergy Immunol..

[4] Doeing D.C., Solway J. (2013). Airway smooth muscle in the pathophysiology and treatment of asthma. J. Appl. Physiol..

[5] Kume H. (2021). Role of airway smooth muscle in inflammation related to asthma and COPD. Adv. Exp. Med. Biol..

[6] Shimoda T., Obase Y., Kishikawa R., Iwanaga T. (2016). Influence of cigarette smoking on airway inflammation and inhaled corticosteroid treatment in patients with asthma. Allergy Asthma Proc..

[7] Tommola M., Ilmarinen P., Tuomisto L.E., Haanpää J., Kankaanranta T., Niemelä O., Kankaanranta H. (2016). The effect of smoking on lung function: A clinical study of adult-onset asthma. Eur. Respir. J..

[8] Korsbæk N., Landt E.M., Dahl M. (2021). Second-hand smoke exposure associated with risk of respiratory symptoms, asthma, and COPD in 20,421 adults from the General Population. J. Asthma Allergy.

[9] Keogan S., Alonso T., Sunday S., Tigova O., Fernández E., López M.J., Gallus S., Semple S., Tzortzi A., Boffi R. (2021). Lung function changes in patients with chronic obstructive pulmonary disease (COPD) and asthma exposed to secondhand smoke in outdoor areas. J. Asthma.

[10] Coogan P.F., Castro-Webb N., Yu J., O’Connor G.T., Palmer J.R., Rosenberg L. (2015). Active and passive smoking and the incidence of asthma in the Black Women’s Health Study. Am. J. Respir. Crit. Care Med..

[11] Van der Toorn M., Rezayat D., Kauffman H.F., Bakker S.J.L., Gans R.O.B., Koëter G.H., Choi A.M.K., Van Oosterhout A.J.M., Slebos D.-J. (2009). Lipid-soluble components in cigarette smoke induce mitochondrial production of reactive oxygen species in lung epithelial cells. Am. J. Physiol. Lung. Cell Mol. Physiol..

[12] Zhou G., Xiao W., Xu C., Hu Y., Wu X., Huang F., Lu X., Shi C., Wu X. (2016). Chemical constituents of tobacco smoke induce the production of interleukin-8 in human bronchial epithelium, 16HBE cells. Tob. Induc. Dis..

[13] Ong J., van den Berg A., Faiz A., Boudewijn I.M., Timens W., Vermeulen C.J., Oliver B.G., Kok K., Terpstra M.M., van den Berge M. (2019). Current smoking is associated with decreased expression of miR-335–5p in parenchymal lung fibroblasts. Int. J. Mol. Sci..

[14] Mirra D., Cione E., Spaziano G., Esposito R., Sorgenti M., Granato E., Cerqua I., Muraca L., Iovino P., Gallelli L. (2022). Circulating microRNAs expression profile in lung inflammation: A preliminary study. J. Clin. Med..

[15] Cipollina C., Bruno A., Fasola S., Cristaldi M., Patella B., Inguanta R., Vilasi A., Aiello G., La Grutta S., Torino C. (2022). Cellular and molecular signatures of oxidative stress in bronchial epithelial cell models injured by cigarette smoke extract. Int. J. Mol. Sci..

[16] Strzelak A., Ratajczak A., Adamiec A., Feleszko W. (2018). Tobacco smoke induces and alters immune responses in the lung triggering inflammation, allergy, asthma and other lung diseases: A mechanistic review. Int. J. Environ. Res. Public Health.

[17] Alsharairi N.A. (2021). Scutellaria baicalensis and their natural flavone compounds as potential medicinal drugs for the treatment of nicotine-induced non-small-cell lung cancer and asthma. Int. J. Environ. Res. Public Health.

[18] Vonk J.M., Scholtens S., Postma D.S., Moffatt M.F., Jarvis D., Ramasamy A., Wjst M., Omenaas E.R., Bouzigon E., Demenais F. (2017). Adult onset asthma and interaction between genes and active tobacco smoking: The GABRIEL consortium. PLoS ONE.

[19] Uh S.-T., Park J.-S., Koo S.-M., Kim Y.-K., Kim K.U., Kim M.-A., Shin S.-W., Son J.-H., Park H.-W., Shin H.D. (2019). Association of genetic variants of NLRP4 with exacerbation of asthma: The effect of smoking. DNA Cell. Biol.

[20] Losol P., Kim S.H., Ahn S., Lee S., Choi J.-P., Kim Y.-H., Hong S.-J., Kim B.-S., Chang Y.-S. (2021). Genetic variants in the TLR-related pathway and smoking exposure alter the upper airway microbiota in adult asthmatic patients. Allergy.

[21] Kim M.-H., Chang H.S., Lee J.-U., Shim J.-S., Park J.-S., Cho Y.-J., Park C.-S. (2022). Association of genetic variants of oxidative stress responsive kinase 1 (*OXSR1*) with asthma exacerbations in non-smoking asthmatics. BMC Pulm. Med..

[22] Belcaro G., Luzzi R., Cesinaro Di Rocco P., Cesarone M.R., Dugall M., Feragalli B., Errichi B.M., Ippolito E., Grossi M.G., Hosoi M. (2011). Pycnogenol® improvements in asthma management. Panminerva. Med..

[23] Thomson N.C., Spears M. (2005). The influence of smoking on the treatment response in patients with asthma. Curr. Opin. Allergy Clin. Immunol..

[24] Thomson N.C., Shepherd M., Spears M., Chaudhuri R. (2006). Corticosteroid insensitivity in smokers with asthma: Clinicalevidence, mechanisms, and management. Treat Respir. Med..

[25] Chatkin J.M., Dullius C.R. (2016). The management of asthmatic smokers. Asthma Res. Pract..

[26] Duman B., Borekci S., Akdeniz N., Gazioglu S.B., Deniz G., Gemicioglu B. (2022). Inhaled corticosteroids’ effects on biomarkers in exhaled breath condensate and blood in patients newly diagnosed with asthma who smoke. J. Asthma.

[27] Alsharairi N.A. (2019). The effects of dietary supplements on asthma and lung cancer risk in smokers and non-smokers: A review of the literature. Nutrients.

[28] Alsharairi N.A. (2021). Supplements for smoking-related lung diseases. Encyclopedia.

[29] Alsharairi N.A. (2022). Dietary antioxidants and lung cancer risk in smokers and non-smokers. Healthcare.

[30] Santillan A.A., Camargo C.A., Colditz G.A. (2003). A meta-analysis of asthma and risk of lung cancer (United States). Cancer Causes Control..

[31] Rosenberger A., Bickeböller H., McCormack V., Brenner D.R., Duell E.J., Tjønneland A., Friis S., Muscat J.E., Yang P., Wichmann H.E. (2012). Asthma and lung cancer risk: A systematic investigation by the International Lung Cancer Consortium. Carcinogenesis.

[32] Burns J.S., Dockery D.W., Neas L.M., Schwartz J., Coull B.A., Raizenne M., Speizer F.E. (2007). Low dietary nutrient intakes and respiratory health in adolescents. Chest.

[33] Zhang W., Li W., Du J. (2022). Association between dietary carotenoid intakes and the risk of asthma in adults: A cross-sectional study of NHANES, 2007–2012. BMJ Open.

[34] Pearson P.J.K., Lewis S.A., Britton J., Fogarty A. (2004). Vitamin E supplements in asthma: A parallel group randomised placebo controlled trial. Thorax.

[35] Shaheen S.O., Newson R.B., Rayman M.P., Wong A.P.-L., Tumilty M.K., Phillips J.M., Potts J.F., Kelly F.J., White P.T., Burney P.G.J. (2007). Randomised, double blind, placebo-controlled trial of selenium supplementation in adult asthma. Thorax.

[36] Van der Vaart H., Postma D.S., Timens W., Ten Hacken N.H.T. (2004). Acute effects of cigarette smoke on inflammation and oxidative stress: A review. Thorax.

[37] Yadav A.S., Saini M. (2016). Evaluation of systemic antioxidant level and oxidative stress in relation to lifestyle and disease progression in asthmatic patients. J. Med. Biochem..

[38] Bartoli M.L., Novelli F., Costa F., Malagrinò L., Melosini L., Bacci E., Cianchetti S., Dente F.L., Di Franco A., Vagaggini B. (2011). Malondialdehyde in exhaled breath condensate as a marker of oxidative stress in different pulmonary diseases. Mediators Inflamm..

[39] Anes A.B., Nasr H.B., Fetoui H., Bchir S., Chahdoura H., Yacoub S., Garrouch A., Benzarti M., Tabka Z., Chahed K. (2016). Alteration in systemic markers of oxidative and antioxidative status in Tunisian patients with asthma: Relationships with clinical severity and airflow limitation. J. Asthma.

[40] Mak J.C.W., Leung H.C.M., Ho S.P., Law B.K.W., Lam W.K., Tsang K.W.T., Ip M.C.M., Chan-Yeung M. (2004). Systemic oxidative and antioxidative status in Chinese patients with asthma. J. Allergy Clin. Immunol..

[41] Nadeem A., Chhabra S.K., Masood A., Raj H.G. (2003). Increased oxidative stress and altered levels of antioxidants in asthma. J. Allergy Clin. Immunol..

[42] Deveci F., Ilhan N., Turgut T., Akpolat N., Kirkil G., Muz M.H. (2004). Glutathione and nitrite in induced sputum from patients with stable and acute asthma compared with controls. Ann. Allergy Asthma Immunol..

[43] Hillas G., Kostikas K., Mantzouranis K., Bessa V., Kontogianni K., Papadaki G., Papiris S., Alchanatis M., Loukides S., Bakakos P. (2011). Exhaled nitric oxide and exhaled breath condensate pH as predictors of sputum cell counts in optimally treated asthmatic smokers. Respirology.

[44] Michils A., Louis R., Peché R., Baldassarre S., Van Muylem A. (2009). Exhaled nitric oxide as a marker of asthma control in smoking patients. Eur. Respir. J..

[45] Pavlidis S., Takahashi K., Kwong F.N.K., Xie J., Hoda U., Sun K., Elyasigomari V., Agapow P., Loza M., Baribaud F. (2019). “T2-high” in severe asthma related to blood eosinophil, exhaled nitric oxide and serum periostin. Eur. Respir. J..

[46] Chamitava L., Cazzoletti L., Ferrari M., Garcia-Larsen V., Jalil A., Degan P., Fois A.G., Zinellu E., Fois S.S., Pasini A.M.F. (2020). Biomarkers of oxidative stress and inflammation in chronic airway diseases. Int. J. Mol. Sci..

[47] Malinovschi A., Backer V., Harving H., Porsbjerg C. (2012). The value of exhaled nitric oxide to identify asthma in smoking patients with asthma-like symptoms. Respir. Med..

[48] Zietkowski Z., Tomasiak-Lozowska M.M., Skiepko R., Zietkowska E., Bodzenta-Lukaszyk A. (2010). Eotaxin-1 in exhaled breath condensate of stable and unstable asthma patients. Respir. Res..

[49] Krisiukeniene A., Babusyte A., Stravinskaite K., Lotvall J., Sakalauskas R., Sitkauskiene B. (2009). Smoking affects eotaxin levels in asthma patients. J. Asthma.

[50] Rossios C., Pavlidis S., Hoda U., Kuo C.-H., Wiegman C., Russell K., Sun K., Loza M.J., Baribaud F., Durham A.L. (2018). Sputum transcriptomics reveal upregulation of IL-1 receptor family members in patients with severe asthma. J. Allergy Clin. Immunol..

[51] Chalmers G.W., MacLeod K.J., Thomson L., Little S.A., McSharry C., Thomson N.C. (2001). Smoking and airway inflammation in patients with mild asthma. Chest.

[52] Thomson N.C., Chaudhuri R., Spears M., Haughney J., McSharry C. (2015). Serum periostin in smokers and never smokers with asthma. Respir. Med..

[53] Yildiz H., Alp H.H., Sünnetçioğlu A., Ekin S., Çilingir B.M. (2021). Evaluation serum levels of YKL-40, Periostin, and some inflammatory cytokines together with IL-37, a new anti-inflammatory cytokine, in patients with stable and exacerbated asthma. Heart Lung.

[54] Rovina N., Dima E., Gerassimou C., Kollintza A., Gratziou C., Roussos C. (2009). IL-18 in induced sputum and airway hyperresponsiveness in mild asthmatics: Effect of smoking. Respir. Med..

[55] Ham J., Kim J., Sohn K.-H., Park I.-W., Choi B.-W., Chung D.H., Cho S.-H., Kang H.R., Jung J.-W., Kim H.Y. (2022). Cigarette smoke aggravates asthma by inducing memory-like type 3 innate lymphoid cells. Nat. Commun..

[56] Liu T., Wu J., Zhao J., Wang J., Zhang Y., Liu L., Cao L., Liu Y., Dong L. (2015). Type 2 innate lymphoid cells: A novel biomarker of eosinophilic airway inflammation in patients with mild to moderate asthma. Respir. Med..

[57] Shimoda T., Obase Y., Kishikawa R., Iwanaga T. (2015). Serum high-sensitivity C-reactive protein can be an airway inflammation predictor in bronchial asthma. Allergy Asthma Proc..

[58] Halvani A., Tahghighi F., Nadooshan H.H. (2012). Evaluation of correlation between airway and serum inflammatory markers in asthmatic patients. Lung. India.

[59] Mikus M.S., Kolmert J., Andersson L.I., Östling J., Knowles R.G., Gómez C., Ericsson M., Thörngren J.-O., Khoonsari P.E., Dahlén B. (2022). Plasma proteins elevated in severe asthma despite oral steroid use and unrelated to Type-2 inflammation. Eur. Respir. J..

[60] Takahashi K., Pavlidis S., Kwong F.N.K., Hoda U., Rossios C., Sun K., Loza M., Baribaud F., Chanez P., Fowler S.J. (2018). Sputum proteomics and airway cell transcripts of current and ex-smokers with severe asthma in U-BIOPRED: An exploratory analysis. Eur. Respir. J..

[61] Emma R., Bansal A.T., Kolmert J., Wheelock C.E., Dahlen S.-E., Loza M.J., De Meulder B., Lefaudeux D., Auffray C., Dahlen B. (2018). Enhanced oxidative stress in smoking and ex-smoking severe asthma in the U-BIOPRED cohort. PLoS ONE.

[62] Thomson N.C., Chaudhuri R., Heaney L.G., Bucknall C., Niven R.M., Brightling C.E., Menzies-Gow A.N., Mansur A.H., McSharry C. (2013). Clinical outcomes and inflammatory biomarkers in current smokers and exsmokers with severe asthma. J. Allergy Clin. Immunol..

[63] Giovannelli J., Chérot-Kornobis N., Hulo S., Ciuchete A., Clément G., Amouyel P., Matran R., Dauchet L. (2016). Both exhaled nitric oxide and blood eosinophil count were associated with mild allergic asthma only in non-smokers. Clin. Exp. Allergy.

[64] Kostikas K., Papaioannou A.I., Tanou K., Giouleka P., Koutsokera A., Minas M., Papiris S., Gourgoulianis K.I., Taylor D.R., Loukides S. (2011). Exhaled NO and exhaled breath condensate pH in the evaluation of asthma control. Respir. Med..

[65] Chu S., Ma L., Wei J., Wang J., Xu Q., Chen M., Jiang M., Luo M., Wu J., Mai L. (2021). Smoking status modifies the relationship between Th2 biomarkers and small airway obstruction in asthma. Can. Respir. J..

[66] Nerpin E., Ferreira D.S., Weyler J., Schlunnsen V., Jogi R., Semjen C.R., Gislasson T., Demoly P., Heinrich J., Nowak D. (2021). Bronchodilator response and lung function decline: Associations with exhaled nitric oxide with regard to sex and smoking status. World Allergy Organ. J..

[67] Cianchetti S., Cardini C., Puxeddu I., Latorre M., Bartoli M.L., Bradicich M., Dente F., Bacci E., Celi A., Paggiaro P. (2019). Distinct profile of inflammatory and remodelling biomarkers in sputum of severe asthmatic patients with or without persistent airway obstruction. World Allergy Organ. J..

[68] Timoneda J., Rodríguez-Fernández L., Zaragozá R., Marín M.P., Cabezuelo M.T., Torres L., Viña J.R., Barber T. (2018). Vitamin A deficiency and the lung. Nutrients.

[69] Defnet A.E., Shah S.D., Huang W., Shapiro P., Deshpande D.A., Kane M.A. (2021). Dysregulated retinoic acid signaling in airway smooth muscle cells in asthma. FASEB J..

[70] Druilhe A., Zahm J.-M., Benayoun L., El Mehdi D., Grandsaigne M., Dombret M.-C., Mosnier I., Feger B., Depondt J., Aubier M. (2008). Epithelium expression and function of retinoid receptors in asthma. Am. J. Respir. Cell. Mol. Biol..

[71] Day R.M., Lee Y.H., Park A.-M., Suzuki Y.J. (2006). Retinoic acid inhibits airway smooth muscle cell migration. Am. J. Respir. Cell. Mol. Biol..

[72] Callaghan P.J., Rybakovsky E., Ferrick B., Thomas S., Mullin J.M. (2020). Retinoic acid improves baseline barrier function and attenuates TNF-α-induced barrier leak in human bronchial epithelial cell culture model, 16HBE 14o. PLoS ONE.

[73] Wu J., Zhang Y., Liu Q., Zhong W., Xia Z. (2013). All-trans retinoic acid attenuates airway inflammation by inhibiting Th2 and Th17 response in experimental allergic asthma. BMC Immunol..

[74] Takamura K., Nasuhara Y., Kobayashi M., Betsuyaku T., Tanino Y., Kinoshita I., Yamaguchi E., Matsukura S., Schleimer R.P., Nishimura M. (2004). Retinoic acid inhibits interleukin-4-induced eotaxin production in a human bronchial epithelial cell line. Am. J. Physiol. Lung. Cell. Mol. Physiol..

[75] Kurie J.M., Lotan R., Lee J.J., Lee J.S., Morice R.C., Liu D.D., Xu X.-C., Khuri F.R., Ro J.Y., Hittelman W.N. (2003). Treatment of former smokers with 9-*cis*-retinoic acid reverses loss of retinoic acid receptorbeta expression in the bronchial epithelium: Results from a randomized placebo-controlled trial. J. Natl. Cancer. Inst..

[76] Frankenberger M., Heimbeck I., Möller W., Mamidi S., Kaßner G., Pukelsheim K., Wjst M., Neiswirth M., Kroneberg P., Lomas D. (2009). Inhaled all-trans retinoic acid in an individual with severe emphysema. Eur. Respir. J..

[77] Saini R.K., Prasad P., Lokesh V., Shang X., Shin J., Keum Y.-S., Lee J.-H. (2022). Carotenoids: Dietary sources, extraction, encapsulation, bioavailability, and health benefits-A review of recent advancements. Antioxidants.

[78] Park H.S., Kim S.R., Kim J.O., Lee Y.C. (2010). The roles of phytochemicals in bronchial asthma. Molecules.

[79] Arora A., Willhite C.A., Liebler D.C. (2001). Interactions of beta-carotene and cigarette smoke in human bronchial epithelial cells. Carcinogenesis.

[80] Prakash P., Liu C., Hu K.-Q., Krinsky N.I., Russell R.M., Wang X.-D. (2004). Beta-carotene and beta-apo-14′-carotenoic acid prevent the reduction of retinoic acid receptor beta in benzo[a]pyrene-treated normal human bronchial epithelial cells. J. Nutr..

[81] Hazlewood L.C., Wood L.G., Hansbro P.M., Foster P.S. (2011). Dietary lycopene supplementation suppresses Th2 responses and lung eosinophilia in a mouse model of allergic asthma. J. Nutr. Biochem..

[82] Lian F., Wang X.-D. (2008). Enzymatic metabolites of lycopene induce Nrf2-mediated expression of phase II detoxifying/antioxidant enzymes in human bronchial epithelial cells. Int. J. Cancer.

[83] Chiaverelli R.A., Hu K.-Q., Liu C., Lim J.Y., Daniels M.S., Xia H., Mein J., von Lintig J., Wang X.-D. (2023). β-Cryptoxanthin attenuates cigarette-smoke-induced lung lesions in the absence of carotenoid cleavage enzymes (BCO1/BCO2) in mice. Molecules.

[84] Liu C., Bronson R.T., Russell R.M., Wang X.-D. (2011). β-Cryptoxanthin supplementation prevents cigarette smoke-induced lung inflammation, oxidative damage, and squamous metaplasia in ferrets. Cancer Prev. Res..

[85] Allen S., Britton J.R., Leonardi-Bee J.A. (2009). Association between antioxidant vitamins and asthma outcome measures: Systematic review and meta-analysis. Thorax.

[86] Tecklenburg S.L., Mickleborough T.D., Fly A.D., Bai Y., Stager J.M. (2007). Ascorbic acid supplementation attenuates exercise-induced bronchoconstriction in patients with asthma. Respir. Med..

[87] Chang H.-H., Chen C.-S., Lin J.-Y. (2009). High dose vitamin C supplementation increases the Th1/Th2 cytokine secretion ratio, but decreases eosinophilic infiltration in bronchoalveolar lavage fluid of ovalbumin-sensitized and challenged mice. J. Agric. Food Chem..

[88] Zhu W., Cromie M.M., Cai Q., Lv T., Singh K., Gao W. (2014). Curcumin and vitamin E protect against adverse effects of benzo[a]pyrene in lung epithelial cells. PLoS ONE.

[89] Hoskins A., Roberts J.L., Milne G., Choi L., Dworski R. (2012). Natural-source d-α-tocopheryl acetate inhibits oxidant stress and modulates atopic asthma in humans In Vivo. Allergy.

[90] Wiser J., Alexis N.E., Jiang Q., Wu W., Robinette C., Roubey R., Peden D.B. (2008). In vivo gamma-tocopherol supplementation decreases systemic oxidative stress and cytokine responses of human monocytes in normal and asthmatic subjects. Free Radic. Biol. Med..

[91] Hernandez M.L., Wagner J.G., Kala A., Mills K., Wells H.B., Alexis N.E., Lay J.C., Jiang Q., Zhang H., Zhou H. (2013). Vitamin E, γ-tocopherol, reduces airway neutrophil recruitment after inhaled endotoxin challenge in rats and in healthy volunteers. Free Radic. Biol. Med..

[92] Burbank A.J., Duran C.G., Pan Y., Burns P., Jones S., Jiang Q., Yang C., Jenkins S., Wells H., Alexis N. (2018). Gamma tocopherol-enriched supplement reduces sputum eosinophilia and endotoxin-induced sputum neutrophilia in volunteers with asthma. J. Allergy Clin. Immunol..

[93] Talati M., Meyrick B., Peebles Jr R.S., Davies S.S., Dworski R., Mernaugh R., Mitchell D., Boothby M., Roberts L.J., Sheller J.R. (2006). Oxidant stress modulates murine allergic airway responses. Free Radic. Biol. Med..

[94] Quoc Q.L., Bich T.C.T., Kim S.-H., Park H.-S., Shin Y.S. (2021). Administration of vitamin E attenuates airway inflammation through restoration of Nrf2 in a mouse model of asthma. J. Cell Mol. Med..

[95] Li J., Li L., Chen H., Chang Q., Liu X., Wu Y., Wei C., Li R., Kwan J.K.C., Yeung K.L. (2014). Application of vitamin E to antagonize SWCNTs-induced exacerbation of allergic asthma. Sci. Rep..

[96] Mabalirajan U., Aich J., Leishangthem G.D., Sharma S.K., Dinda A.K., Ghosh B. (2009). Effects of vitamin E on mitochondrial dysfunction and asthma features in an experimental allergic murine model. J. Appl. Physiol..

[97] McCary C.A., Abdala-Valencia H., Berdnikovs S., Cook-Mills J.M. (2011). Supplemental and highly elevated tocopherol doses differentially regulate allergic inflammation: Reversibility of α-tocopherol and γ-tocopherol’s effects. J. Immunol..

[98] Hall S.C., Agrawal D.K. (2017). Vitamin D and bronchial asthma: An overview of data from the past 5 years. Clin. Ther..

[99] Pfeffer P.E., Lu H., Mann E.H., Chen Y.-H., Ho T.-R., Cousins D.J., Corrigan C., Kelly F.J., Mudway I.S., Hawrylowicz C.M. (2018). Effects of vitamin D on inflammatory and oxidative stress responses of human bronchial epithelial cells exposed to particulate matter. PLoS ONE.

[100] Ramos-Martínez E., López-Vancell M.R., Fernández de Córdova-Aguirre J.C., Rojas-Serrano J., Chavarría A., Velasco-Medina A., Velázquez-Sámano G. (2018). Reduction of respiratory infections in asthma patients supplemented with vitamin D is related to increased serum IL-10 and IFNγ levels and cathelicidin expression. Cytokine.

[101] Adam-Bonci T.-I., Bonci E.-A., Pârvu A.-E., Herdean A.-I., Moț A., Taulescu M., Ungur A., Pop R.-M., Bocșan C., Irimie A. (2021). Vitamin D supplementation: Oxidative stress modulation in a mouse model of ovalbumin-induced acute asthmatic airway inflammation. Int. J. Mol. Sci..

[102] Huang C., Peng M., Tong J., Zhong X., Xian J., Zhong L., Deng J., Huang Y. (2022). Vitamin D ameliorates asthma-induced lung injury by regulating HIF-1α/Notch1 signaling during autophagy. Food Sci. Nutr..

[103] Fischer K.D., Hall S.C., Agrawal D.K. (2016). Vitamin D supplementation reduces induction of epithelial-mesenchymal transition in allergen sensitized and challenged mice. PLoS ONE.

[104] Gorman S., Weeden C.E., Tan D.H.W., Scott N.M., Hart J., Foong R.E., Mok D., Stephens N., Zosky G., Hart P.H. (2013). Reversible control by vitamin D of granulocytes and bacteria in the lungs of mice: An ovalbumin-induced model of allergic airway disease. PLoS ONE.

[105] Taher Y.A., van Esch B.C.A.M., Hofman G.A., Henricks P.A.J., van Oosterhout A.J.M. (2008). 1alpha,25-dihydroxyvitamin D3 potentiates the beneficial effects of allergen immunotherapy in a mouse model of allergic asthma: Role for IL-10 and TGF-beta. J. Immunol..

[106] Agrawal T., Gupta G.K., Agrawal D.K. (2013). Vitamin D supplementation reduces airway hyperresponsiveness and allergic airway inflammation in a murine model. Clin. Exp. Allergy.

[107] Zhang H., Yang N., Wang T., Dai B., Shang Y. (2018). Vitamin D reduces inflammatory response in asthmatic mice through HMGB1/TLR4/NF-κB signaling pathway. Mol. Med. Rep..

[108] Ekmekci O.P., Donma O., Sardoğan E., Yildirim N., Uysal O., Demirel H., Demir T. (2004). Iron, nitric oxide, and myeloperoxidase in asthmatic patients. Biochemistry.

[109] Narula M.K., Ahuja G.K., Whig J., Narang A.P.S., Soni R.K. (2007). Status of lipid peroxidation and plasma iron level in bronchial asthmatic patients. Indian J. Physiol. Pharmacol..

[110] Han F., Li S., Yang Y., Bai Z. (2021). Interleukin-6 promotes ferroptosis in bronchial epithelial cells by inducing reactive oxygen species-dependent lipid peroxidation and disrupting iron homeostasis. Bioengineered.

[111] Kuribayashi K., Iida S.-I., Nakajima Y., Funaguchi N., Tabata C., Fukuoka K., Fujimori Y., Ihaku D., Nakano T. (2015). Suppression of heme oxygenase-1 activity reduces airway hyperresponsiveness and inflammation in a mouse model of asthma. J. Asthma.

[112] Ghio A.J., Hilborn E.D. (2017). Indices of iron homeostasis correlate with airway obstruction in an NHANES III cohort. Int. J. Chron. Obstruct. Pulmon. Dis..

[113] Ghio A.J., Hilborn E.D., Stonehuerner J.G., Dailey L.A., Carter J.D., Richards J.H., Crissman K.M., Foronjy R.F., Uyeminami D.L., Pinkerton K.E. (2008). Particulate matter in cigarette smoke alters iron homeostasis to produce a biological effect. Am. J. Respir. Crit. Care. Med..

[114] Zajac D. (2021). Mineral micronutrients in asthma. Nutrients.

[115] Fahmy B., Cormier S.A. (2009). Copper oxide nanoparticles induce oxidative stress and cytotoxicity in airway epithelial cells. Toxicol. Vitr..

[116] Park J.-W., Lee I.-C., Shin N.-R., Jeon C.-M., Kwon O.-K., Ko J.-W., Kim J.-C., Oh S.-R., Shin I.-S., Ahn K.-S. (2016). Copper oxide nanoparticles aggravate airway inflammation and mucus production in asthmatic mice via MAPK signaling. Nanotoxicology.

[117] Sagdic A., Sener O., Bulucu F., Karadurmus N., Özel H.E., Yamanel L., Tasci C., Naharci I., Ocal R. (2011). Aydin Oxidative stress status and plasma trace elements in patients with asthma or allergic rhinitis. Allergol. Immunopathol..

[118] Guo C.-H., Liu P.-J., Hsia S., Chuang C.-J., Chen P.-C. (2011). Role of certain trace minerals in oxidative stress, inflammation, CD4/CD8 lymphocyte ratios and lung function in asthmatic patients. Ann. Clin. Biochem..

[119] Mao S., Wu L., Shi W. (2018). Association between trace elements levels and asthma susceptibility. Respir. Med..

[120] Richter M., Cantin A.M., Beaulieu C., Cloutier A., Larivée P. (2003). Zinc chelators inhibit eotaxin, RANTES, and MCP-1 production in stimulated human airway epithelium and fibroblasts. Am. J. Physiol. Lung Cell Mol. Physiol..

[121] Lang C., Murgia C., Leong M., Tan L.-W., Perozzi G., Knight D., Ruffin R., Zalewski P. (2007). Anti-inflammatory effects of zinc and alterations in zinc transporter mRNA in mouse models of allergic inflammation. Am. J. Physiol. Lung. Cell. Mol. Physiol..

[122] Lu H., Xin Y., Tang Y., Shao G. (2012). Zinc suppressed the airway inflammation in asthmatic rats: Effects of zinc on generation of eotaxin, MCP-1, IL-8, IL-4, and IFN-γ. Biol. Trace. Elem. Res..

